# Identification of novel components of the Ced and Ups systems in *Saccharolobus islandicus* REY15A

**DOI:** 10.1002/mlf2.12163

**Published:** 2025-02-23

**Authors:** Pengju Wu, Mengqi Zhang, Yanlu Kou, Shikuan Liang, Jinfeng Ni, Qihong Huang, Yulong Shen

**Affiliations:** ^1^ State Key Laboratory of Microbial Technology CRISPR and Archaea Biology Research Center, Microbial Technology Institute, Shandong University Qingdao China

**Keywords:** *Crenarchaea*, Ced, chromosomal DNA exchange, DNA damage response, Ups

## Abstract

In *Sulfolobales* cells, transcription of the Ups (UV‐inducible pili of *Sulfolobus*) and Ced (Crenarchaeal system for exchange of DNA) genes is highly induced by DNA damage, and the two systems play key roles in pili‐mediated cell aggregation and chromosomal DNA import, respectively. Ups is composed of UpsA, UpsB, UpsE, and UpsF, while Ced is composed of CedA, CedA1, CedA2, and CedB. So far, how DNA is transported by these systems is far from clear. Here, we report three novel components of the Ced and Ups systems in *Saccharolobus islandicus* REY15A, CedD (SiRe_1715) and CedE (SiRe_2100), paralogs of CedB and CedA, and UpsC (SiRe_1957), a paralog of UpsA/UpsB. We developed a DNA import and export assay method, by which we revealed that CedD, CedE, and UpsC are essential for DNA import, while CedE and UpsC are also involved in DNA export together with CedA1 and Ups. Microscopic analysis revealed that *upsC* is involved in cell aggregation like other Ups genes. In addition, we found that *cedB* and *cedD* co‐occur in the Crenarchaeal genomes that lack *virB4*, an essential component of type IV secretion system. Interestingly, CedB and CedD share homology to different parts of VirB4 N‐terminal domain and form stable homo‐oligomers in vitro. Collectively, our results indicate that CedD, CedE, and UpsC are integral components of the Ced and Ups systems in *Sulfolobales*.

## Impact statement

In *Sulfolobales*, the Ced (Crenarchaeal system for exchange of DNA) and Ups (UV‐inducible pili of *Sulfolobus*) systems are responsible for chromosomal DNA exchange to repair DNA damage. Two novel components of the Ced system, CedD and CedE, and a new pilin subunit of the Ups system, UpsC, are identified. Our results support that Ced is a T4SS‐like system and functions in both chromosomal DNA export and import. This study provides new insights into the composition and function of the Ced and Ups systems.

## INTRODUCTION

Horizontal gene transfer (HGT) is essential for the adaptation of life to environments. In archaea, HGT occurs through the universal pathways, natural transformation, conjugation, and transduction^1^, or by archaea‐specific mechanisms, such as extracellular vesicle‐mediated DNA transfer in *Thermococcales* and *Sulfolobales* ^2^, ^3^ and chromosomal DNA exchange in *Haloarchaea* and *Sulfolobales* ^4^, ^5^. In the hyperthermophilic *Crenarchaea Sulfolobales*, inter‐cellular chromosomal recombination frequency is 10^−4^–10^−5^, much higher than the spontaneous mutation rate (1–3 × 10^−7^), suggesting that *Sulfolobales* cells utilize efficient DNA transfer to overcome frequently occurring DNA damage at high temperatures^6^, ^7^. Furthermore, it is established that chromosomal DNA exchange in *Sulfolobales* is stimulated by UV irradiation, and the Ups (UV‐inducible pili of *Sulfolobus*) system, which mediates cell aggregation, is indispensable for DNA exchange^8^, ^9^. A system called Ced (Crenarchaeal system for exchange of DNA) was found to be involved in DNA import^10^. However, how DNA is transported by the Ced and Ups systems remains unclear.

Ced is composed of four transmembrane (TM) proteins, CedA, CedA1, CedA2, and CedB, in *Sulfolobus acidocaldarius* ^10^. CedA and CedB are bacterial VirB6 and VirB4 homologs, respectively, and function in DNA import but not export^10^. CedA1 and CedA2 are composed of only two hydrophobic helixes and their functions in DNA transport are unclear. Recently, it has been reported that the CedA1 homolog from *Aeropyrum pernix* K1 forms T4SS (Type IV secretion system)‐like pili, suggesting that CedA1 could be a homolog of VirB2 (bacterial T‐pilin) and function as a conduit for DNA transport^11^. T4SS generally contains VirB2‐VirB11 and VirD4 components^12^, ^13^, ^14^. The main function of T4SS is to export conjugative DNA or effectors^15^, ^16^. During conjugation, double‐stranded conjugative plasmid is first processed by a relaxase complex to form single‐stranded DNA (ssDNA), which is then delivered to ATP‐powered components VirD4, VirB11, and VirB4 sequentially^17^, ^18^. DNA export machinery VirB2‐VirB11 complex is composed of IMCC (inner membrane core complex), OMCC (outer membrane core complex), and stalk which connects IMCC and OMCC^13^. ssDNA is exported by the VirB4 ATPase ring which is composed of six VirB4 dimers^13^. Then, the ssDNA is delivered through the transmembrane channel formed by VirB6 and enters T‐pili formed by VirB2^19^, ^20^, ^21^, ^22^. In rare cases, the T4SS system (such as the ComB system in *Helicobacter Pylori*) is used to uptake DNA^23^. It seems that *Sulfolobales* cells apply a simplified T4SS system for chromosomal DNA transport. So far, only the homologs of VirB2, VirB4, and VirB6 have been found in the Ced system^11^. This raises a question about how DNA import is fulfilled by the archaeal Ced system and if there are additional components of this system.

Different from archaeal conjugation in which conjugative plasmids can even be transferred from order to order (particularly *Thermococcales* to *Desulfurococcales*)^24^, chromosomal DNA exchange in *Sulfolobales* is species‐specific^8^. The specific recognition is mediated by Ups, one of Type IV pili (T4Ps)^25^. In *Saccharolobus islandicus* REY15A, there are four kinds of T4Ps: archaellum, adhesion pili, Ups, and putative bindosome^26^, ^27^, ^28^, ^29^. Only Ups is DNA damage inducible and mediates cell aggregation^29^. Ups components are encoded by a conserved *ups* operon, the genes in which are arranged as *upsX*‐*upsE*‐*upsF*‐*upsA*‐*upsB* in all *Sulfolobales* except *Acidianus*, in which the *upsF*‐*upsA*‐*upsB* are missing^10^, ^30^. UpsX is a membrane protein with unclarified function, and deletion of *upsX* did not affect cell aggregation^30^. UpsE and UpsF function as pilin assembly ATPase and platform, respectively, while UpsA and UpsB are pilin subunits forming pilus which mediates species‐specific aggregation by recognizing different glycosylation patterns^25^, ^30^.

Interestingly, in addition to Ced and Ups genes, many genes are DNA damage inducible but their functions have not been characterized^31^, ^32^, ^33^, ^34^. Here, we report the identification and bioinformatic analysis of three new components designated as CedD, CedE, and UpsC of the Ced and Ups systems. By chromosomal DNA exchange assay and cell aggregation analysis, we found that CedD is only involved in DNA import, probably by forming a DNA translocation channel with CedB, and CedE is involved in both DNA export and import, while UpsC is a new pilin subunit essential for cell aggregation.

## RESULTS

### Identification of putative genes involved in DNA transfer

**Figure 1:**
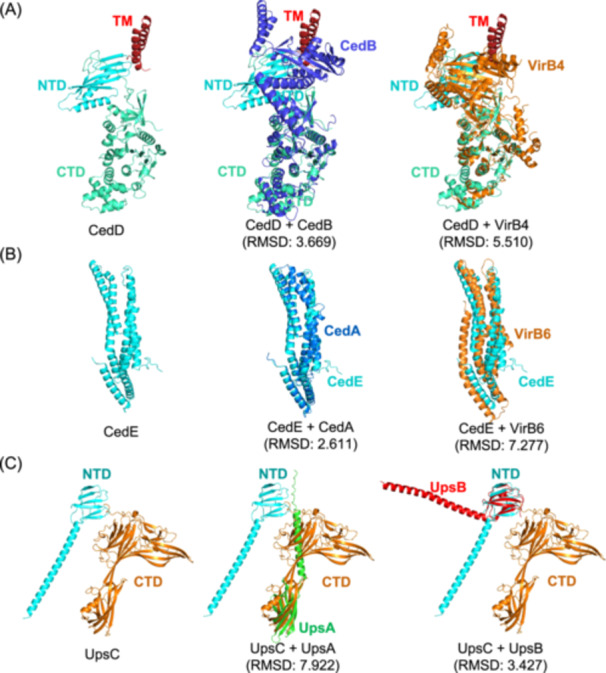
Structures of CedD, CedE, and UpsC and their superposition to the corresponding homologs. (A) Strutures of CedD (left) and its superposition to CedB (middle) and bacterial VirB4 (right). (B) Structures of CedE (left) and its superposition to CedA (middle) and bacterial VirB6 (right). (C) Structures of UpsC (left) and its superposition to UpsA (middle) and UpsB (right). The structures of CedA, CedB, CedD, CedE, UpsA, UpsB, and UpsC were predicted using AlphaFold server and the structures of VirB4 and VirB6 from R388 plasmid were generated from 7o41 and 7o3v of PDB, respectively. Superposition was conducted by PyMOL using Cealign. The root mean square deviation (RMSD) values are shown. CTD, C‐terminal domain; NTD, N‐terminal domain; TM, transmembrane domain.

The Ced and Ups systems were identified by genetic analysis of highly UV‐inducible genes in *Sulfolobus solfataricus* (now *Saccharolobus solfataricus*) and *S. acidocaldarius* ^10^, ^29^, ^34^. Upon treatment with 4‐nitroquinoline 1‐oxide (NQO), a DNA damage agent, 14 genes with uncharacterized functions were upregulated more than 30‐fold in *Sa. islandicus* REY15A^33^. To find additional factors that participate in DNA transfer, we performed bioinformatic analyses using BLAST, Foldseek, and AFDB clusters with the gene products as queries. The products of three genes, *sire_1715*, *sire_2100*, and *sire_1957*, have homology to the Ced and Ups components, CedB, CedA, and UpsA/UpsB, respectively (Figure 1). Therefore, we focused on these proteins in this study. We named the proteins CedD, CedE, and UpsC, respectively, based on the results from this study (Table S1). CedD is composed of a transmembrane (TM) domain, a functionally unknown N‐terminal domain (NTD), and a C‐terminal HerA/VirB4/CedB‐like ATPase domain (Figure 1A). CedE, a CedA paralog specific to the genus *Saccharolobus*, is composed of five TM helixes (Figures 1B and S1). UpsC is a prepilin domain‐containing protein. Its NTD and a portion of its C‐terminal domain can align with UpsB and UpsA, respectively (Figure 1C). UpsC is conserved in *Sulfolobales* (except for the genus *Acidianus*) like the reported Ups proteins^10^.

To confirm that the transcription of *cedD*, *cedE*, and *upsC* is DNA damage inducible, RT‐qPCR was conducted using samples taken at 0, 3, 6, 12, and 24 h after 100 J/m^2^ UV treatment (Figure S2). At 3 h, the transcriptional levels of *cedD*, *cedE*, and *upsC* were upregulated about 23, 78, and 45 folds, respectively, which were close to those of *cedB*, *cedA*, and *upsA*. The levels slightly decreased at 6 h and 12 h and finally returned to the untreated at 24 h (Figure S2). Given that the three genes are paralogs of the reported Ced and Ups components and are induced by DNA damage at similar levels to their paralogs, we speculate that they are putatively novel Ced and Ups components that play important roles in chromosomal DNA exchange.

### Development of a CRISPR‐Cas‐based chromosomal DNA exchange assay

**Figure 2:**
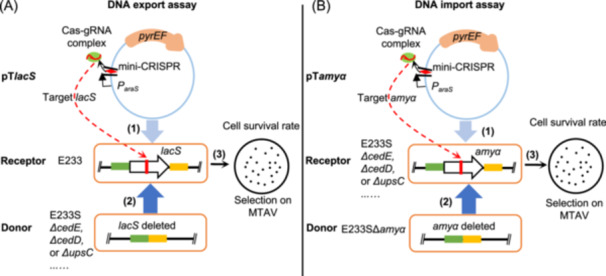
Schematic diagrams showing the developed CRISPR‐Cas‐based chromosomal DNA export and import assays. (A) DNA export assay. Target plasmid pT*lacS* containing a mini‐CRISPR against *lacS* is electro‐transformed into E233 containing *lacS* and incubated at 75°C for 1 h (1). Then, cells are mixed with each of the donor strains, E233S (*lacS* deleted) and E233S derivatives, Δ*cedD*, Δ*cedE*, and Δ*upsC*, etc. The mixture is incubated in inducible medium MTAV at 75°C for 2 h when donor chromosomal DNA is transferred into receptor cells (2). In receptor cell, the *lacS* gene is targeted by the endogenous CRISPR‐Cas system resulting in double‐strand breaks (DSBs). The receptor cells can be rescued by homologous recombinational repair with chromosomal DNA (with *lacS* deletion) that is exported from the donor cells and survive on MTAV plates (3). Donor cell cannot survive due to lack of uracil synthesis ability. (B) DNA import assay. Similar strategy is used for DNA import assay in which *amyα* is used as target. The DNA export and import efficiency is defined as colony forming unit (CFU) on the plates after mixing with the donor cells divided by CFU of the correspongding receptor strain using a non‐targeting plasmid. Finally, the relative DNA export and import efficiency is normalized to the efficiency of E233S as 100%.

To discriminate whether a gene is involved in DNA export or import, we developed a CRISPR‐Cas‐based chromosomal DNA export and import assay modified from a DNA import assay method reported previously^35^ (Figure 2). Briefly, a receptor strain carrying a mini‐CRISPR plasmid is constructed. The CRISPR RNA of the receptor cell is arabinose inducible, and a site‐specific double‐strand break (DSB) can be generated when the cell is cultivated in arabinose‐containing medium. After mixing with a donor cell which carries a non‐target loci, the receptor cells can be rescued by chromosomal DNA exchange between the donor and receptor cells and subsequent homologous recombination. For the DNA export assay, the *lacS* gene was selected as a target and plasmid pT*lacS* was used as a mini‐CRISPR plasmid (Figure 2A). For the DNA import assay, the *amyα* gene in E233S was selected as a target and pT*amyα* was used as a mini‐CRISPR plasmid (Figure 2B). DNA export or import efficiency is calculated as colony forming unit (CFU) on the plates after mixing with the donor cells divided by CFU of the receptor strain transformed using a non‐target plasmid. Relative DNA export or import efficiency is calculated as the efficiency of the test strain with gene deletion divided by that of the control E233S.

To verify this assay, we tested the colony formation of E233 transformed with pT*lacS* (E233::pT*lacS*) and E233S transformed with pT*amyα* (E233S::pT*amyα*) without donor strains. As shown in Figure S3, no colonies or only one colony formed on these plates, while hundreds of colonies formed with the corresponding donor strains (E233::pT*lacS*×E233S and E233S::pT*amyα*×Δ*amyα*), suggesting that targeting with pT*lacS* and pT*amyα* is effective and efficient (close to 100%) and the wild‐type cells E233S is highly efficient in both DNA export and import. In addition, it could be excluded that donor strains obtained plasmids by natural transformation, since few colonies grew on plates when E233S cells were directly mixed with pT*lacS* or Δ*amyα* mixed with pT*amyα* (Figure S3).

**Figure 3:**
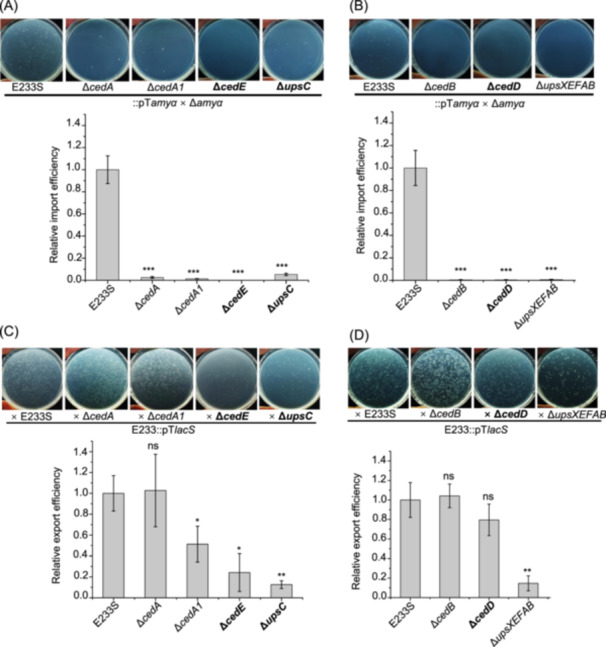
CedE and UpsC are involved in both DNA import and export while CedD is only involved in DNA import. (A, B) Chromosomal DNA import efficiency of Δ*cedA*, Δ*cedA1*, Δ*cedE*, and Δ*upsC* (A) and Δ*cedB*, Δ*cedD*, and Δ*upsXEFAB* (deletion of the operon for the Ups pili) (B). (C, D) Chromosomal DNA export efficiency of Δ*cedA*, Δ*cedA1*, Δ*cedE*, and Δ*upsC* (C) and Δ*cedB*, Δ*cedD*, and Δ*upsXEFAB* (D). Representative plates are shown at the upper panels and quantification of the data is shown at the lower panels. The average relative export efficiency is normalized to that of E233S (*n* = 3 independent experiments, ±SD). The significance was calculated by independent samples *t*‐test using SPSS (ns, no significance; *p ≤ 0.05; **p ≤ 0.005; ***p ≤ 0.001).

Then, we tested the DNA export and import efficiency of knockout strains of the previously reported Ced and Ups components individually. Consistent with the previous report, *cedA* and *cedB* deletion only resulted in DNA import efficiency decrease, while deletion of Ups pili operon *upsXEFAB* abolished both DNA export and import, possibly because cell aggregation was abolished (Figure 3). These results confirmed that our established assay system is effective for testing DNA export and import in *Sa. islandicus*.

### CedE and UpsC are critical for both DNA export and import while CedD is only essential for DNA import

Next, deletion strains of the three putative Ced and Ups components were constructed and their chromosomal DNA export and import ability were examined. We found that Δ*cedD*, Δ*cedE*, and Δ*upsC* lost DNA import ability, the same as Δ*cedA*, Δ*cedA1*, Δ*cedB*, and Δ*upsXEFAB* (Figure 3A,B), suggesting that these three new proteins play essential roles in DNA import, probably synergistically with CedA, CedA1, CedB, and Ups. Interestingly, the DNA export efficiency of Δ*cedE* and Δ*upsC* decreased to about 30% and 10% of that for E233S, respectively. The DNA export efficiency of Δ*cedA1* also decreased to about 50%; however, those of Δ*cedD*, Δ*cedA*, and Δ*cedB* did not change compared with E233S (Figure 3C,D). These results suggest that CedE and UpsC, together with CedA1, are critical for both DNA export and import while CedD is only essential for DNA import.

### CedD and CedB can constitute a complete VirB4‐like NTD and co‐occur in Crenarchaeal genomes

**Figure 4:**
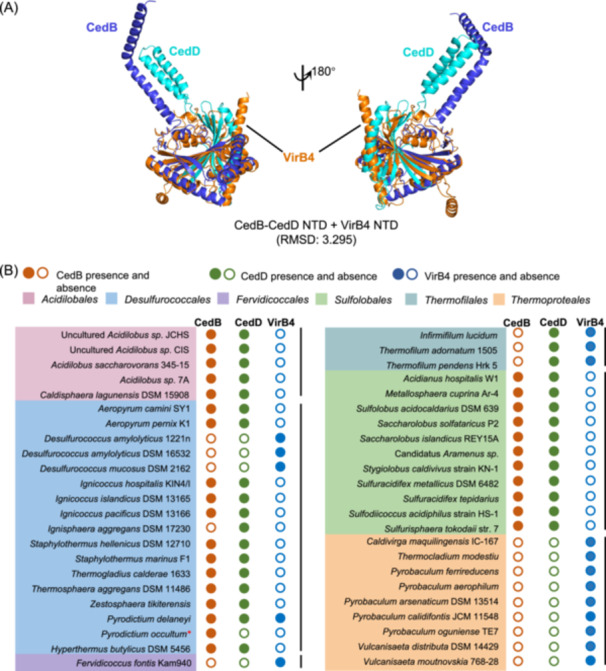
Structural and genomic analysis of CedB/CedD and VirB4 in *Crenarchaeota*. (A) The NTDs of CedB and CedD are superposable to VirB4 NTD. The complex structure of CedB and CedD NTDs was predicted by ColabFold and the structure of VirB4 NTD of R388 plasmid was generated using PDB 7o41. The superposition was conducted by PyMOL using Cealign. The RMSD value is shown. (B) Distribution of CedB and CedD is complementary to that of VirB4 in most *Crenarchaeota*. A total of 85 sequences from 46 species of *Crenarchaeota* were analyzed. Filled and empty circles indicate the presence and absence of the homolog in the analyzed species, respectively. Red asterisk indicates a species of which complete genome sequence is not available.

Previously, CedB and CedD were classified as HerA clade proteins with C‐terminal ATPase domains that have common sequence features^36^. We found that their NTDs are structural homologs of the NTD of VirB4 (Figure 4A), another clade of FtsK‐HerA superfamily functioning in DNA export in T4SS^18^. Interestingly, the NTDs of CedB and CedD are able to be superposed to different parts of bacterial VirB4 NTD (Figure 4A), constituting a full hypothetical VirB4 NTD homolog. More interestingly, CedB/CedD and VirB4 are distributed in a complementary pattern in most *Crenarchaea* (Figure 4B). CedB and CedD co‐occur exclusively in *Acidilobales*, *Sulfolobales*, and most *Desulfurococcales*, while VirB4 exists in *Fervidicoccales*, *Thermoproteales*, and *Desulfurococcus* (Figure 4B), although in *Thermofilales* both CedD and VirB4 (but not CedB) are present. The apparently complementary distribution of CedB/CedD and VirB4 implies that CedB and CedD could be equivalent to VirB4. The results also imply that T4SS and T4SS‐like DNA exchange systems are important for life and are therefore conserved in *Crenarchaea*.

Then, we analyzed the phylogenetic relationships of the conserved ATPase domains of CedB, CedD, and VirB4. As shown in Figure S4, CedB, CedD, and VirB4 homologs were clearly divided into three clades. In CedB and CedD clades, homologs from the same orders cluster together, indicating that CedB and CedD possibly evolved from a common ancestor in *Crenarchaea* and diverged at an early evolutionary stage. Supporting this assumption, a VirB4 homolog from *Pyrobaculum calidifontis* clusters with a bacterial VirB4. This homolog could represent the oldest VirB4 in *Crenarchaeota*.

### CedD facilitates the expression of CedB and both form homo‐oligomers in vitro

**Figure 5:**
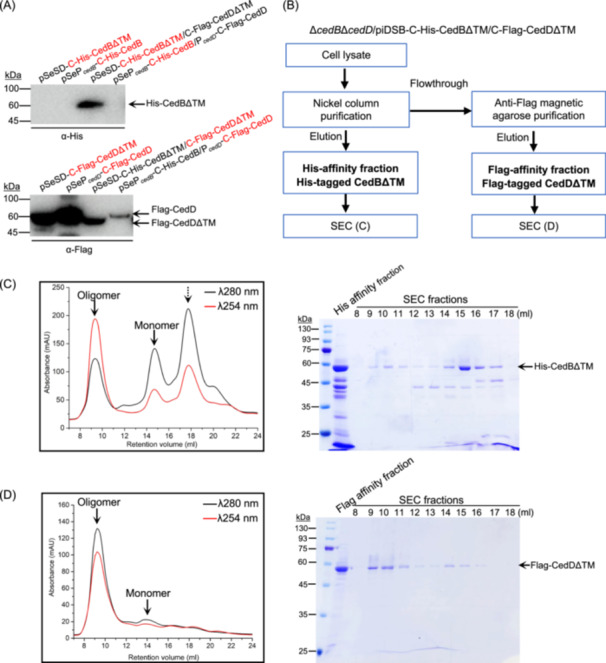
CedD facilitates the expression of CedB and both form homo‐oligomers in vitro. (A) Western blot of samples from different strains. CedB and CedD were detected with anti‐His (upper) and anti‐Flag (lower) antibodies, respectively. Plasmids carried by E233S cells are indicated above. Proteins for detection are highlighted in red. For pSeSD‐based plasmids, D‐arabinose was added for protein expression induction. For native promoter‐based plasmids, DNA damage agent NQO was added for the induction. (B) Schematic diagram showing the procedure for the purification and analysis of His‐tagged CedBΔTM and Flag‐tagged CedDΔTM. (C) Size‐exclusion chromatography (SEC) analysis of nickel column purified His‐tagged CedBΔTM (left) and SDS‐PAGE analysis of the fractions (right). (D) Purification and analysis of Flag‐tagged CedDΔTM. The flowthrough from the nickel column (B) was subjected to anti‐Flag magnetic agarose affinity purification and the elution was analyzed by SEC (left). SDS‐PAGE analysis of the fractions is shown on the right. Lanes 8‐18, fractions (1 ml each) in SEC. Theoretical oligomeric and monomeric peaks of CedBΔTM and CedDΔTM are indicated by solid arrows. Peak of non‐specific protein in (C) is indicated by a dotted arrow.

To probe the mechanism of CedB and CedD in DNA import, we attempted to express and purify CedB and CedD in *Sa. islandicus* E233S. Expression of the full‐length CedB and CedD using strains carrying pSeSD‐based vectors (with D‐arabinose inducible promoter P_*araS*_) was unsuccessful. Hence, we tried to express CedBΔTM and CedDΔTM using P_*araS*_ and the full‐length CedB and CedD using their native promoters. Both CedBΔTM and CedDΔTM could be detected when they were co‐expressed using P_*araS*_, while only full‐length CedD and CedDΔTM were detectable when CedD and CedB proteins were expressed individually (Figure 5A). Next, we co‐expressed His‐tagged CedBΔTM and Flag‐tagged CedDΔTM in E233SΔ*cedB*Δ*cedD* using a strain carrying the piDSB‐based vector, in which proteins together with DSBs were induced by D‐arabinose. However, after His‐tag affinity purification, only CedBΔTM was detectable in the elution fraction, indicating that TM‐deleted CedB and CedD cannot form hetero‐oligomers (Figure S5A). To obtain CedDΔTM, Flag‐tag affinity purification was performed using the flowthrough from previous His‐tag affinity purification (Figure 5B). Interestingly, both His‐CedBΔTM and Flag‐CedDΔTM samples exhibited two peaks in different ratios in size‐exclusion chromatography (SEC) profiles, corresponding to homo‐oligomers and monomers (Figures 5C,D and S5B), respectively. Further SEC analysis of His‐CedBΔTM showed that the oligomer did not convert to monomer and vice versa, suggesting that both oligomeric and monomeric forms of His‐CedBΔTM are stable and its homo‐oligomer formation might require the presence of CedDΔTM (Figure S5C,D). These results suggest that CedD facilitates the expression and homo‐oligomer formation of CedB, although a tight complex of CedB and CedD could not be obtained.

### UpsC functions in cell aggregation in cooperation with other Ups components

**Figure 6:**
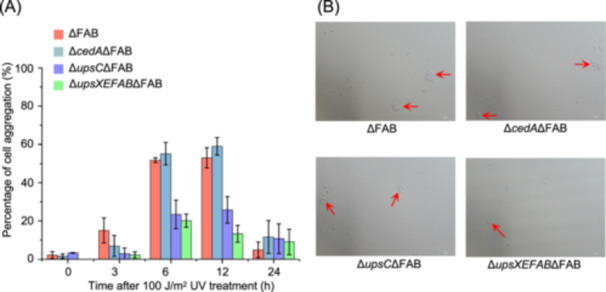
UpsC is involved in UV‐induced cell aggregation, which is independent of the other T4Ps (FAB) archaellum, adhesion pili, and bindosome as well as Ced. (A) Percentages of cell aggregation in different strains at 0, 3, 6, 12, and 24 h after 100 J/m^2^ UV treatment. The values were calculated based on three independent experiments and standard deviation (±SD) was indicated. Cell aggregation was defined as cell clustering with at least three cells. (B) Representative microscopic photos of cells at 6 h after 100 J/m^2^ UV treatment. The aggregated cells are indicated by red arrows.

Because UpsC has structural homology to T4P pilin (Figure 1C) and *upsC* deletion resulted in significant reduction of DNA export and import efficiency (Figure 3), we assumed that UpsC is probably one component of Ups and involved in cell aggregation. To explore the relationship between UpsC and other T4Ps and to investigate whether UpsC is involved in cell aggregation in cooperation with the Ups system, we knocked out genes for T4Ps in *Sa. islandicus* REY15A, including archaellum (F), adhesion pili (A), putative bindosome (B), and Ups pili^26^, ^27^, ^28^, ^29^ (Figure S6), and constructed a series of deletion strains, including ΔFAB in which other T4P systems genes were knocked out, i.e., Δ*cedA*ΔFAB, Δ*upsC*ΔFAB, and Δ*upsXEFAB*ΔFAB. At 6 h after UV treatment, Δ*cedA*ΔFAB exhibited about 50% cell aggregation similar to the control ΔFAB, while cell aggregation ratios of Δ*upsC*ΔFAB and Δ*upsXEFAB*ΔFAB were about 20% (Figure 6A). This result supports that UpsC functions in cell aggregation and cooperates with Ups pili while the Ced system is not involved in cell aggregation during DNA damage response^10^. Moreover, UpsC functions in aggregation normally in ΔFAB, suggesting that UpsC assembly is independent of archaellum, adhesion pili, and bindosome. We also found that cell aggregation was not fully abolished with only three to seven cell clustering together in Δ*upsC*Δ*FAB* or Δ*upsXEFAB*Δ*FAB* (Figure 6A,B). This suggests that other factors may play partial roles in cell contact in *Sa. islandicus* REY15A.

## DISCUSSION

DNA export and uptake mechanisms such as conjugation and natural transformation have been studied in detail in bacteria^17^, ^37^, ^38^. However, DNA exchange in archaea is under investigation and far from clear. This may be due to the existence of diverse DNA transport pathways and the absence of bacterial homologs such as relaxase^1^, ^24^, ^39^. In *Sulfolobales*, the Ced system is involved in DNA import as part of the DNA damage response mechanism^10^. CedA and CedB are homologs of bacterial T4SS components VirB6 and VirB4, respectively^10^, ^11^. CedA1 pili also shares structural similarity to T4SS pili^11^. In this study, we found that CedD and CedE are additional structural homologs of VirB4 and VirB6, respectively (Figures 1A,B and 4A). These results support that the Crenarchaeal specific Ced system is T4SS‐like, suggesting that it could work similarly to bacterial T4SS. These findings and the results from phylogenetic analysis (Figures S1 and S4) imply that Ted (the DNA exchange system in *Thermoproteales*) is the most simplified T4SS while the Ced system in *Sulfolobales* gained additional functionally differentiated paralogs during evolution^11^.

To discriminate DNA export and import process, we developed a CRISPR‐Cas‐based chromosomal DNA exchange assay method (Figure 2). By targeting two protospacers, we could generate DSBs more efficiently at single sites by the endogenous CRISPR‐Cas systems than previously reported^35^. In the DNA import assay, no or only a few colonies grew on Δ*cedB*::pT*amyα*×Δ*amyα* plate, indicating that the plasmid in the receptor cell was not transferred to the donor. This observation also indicates that DNA transport from a defined donor to a receptor can be examined using our method, whereas the chromosomal marker exchange method^10^ assays the combined effects of both import and export. One question remains about how chromosomal and plasmid DNA is discriminated by the donor cells. On the other hand, the receptor cells, in which genomic DSBs are generated, are obtained by electroporation, which possibly makes cells in an abnormal status. Therefore, we attempted to construct a plasmid that could be used to strictly and efficiently induce DSBs by CRISPR‐Cas systems. However, we only obtained piDSB in which the expression of crRNA can be regulated but the targeting efficiency is not 100%. In addition, the export and import efficiency reflects the overall efficiency of DNA processing, export, import, and homologous recombination. Therefore, to more accurately quantify the roles of the DNA transport proteins such as CedB and CedD, homologous recombination efficiency should be evaluated in the deletion strains. Additionally, other methods for conjugation analysis, such as TrIP (transfer DNA immunoprecipitation), a technique used to identify interactions between conjugative DNA and T4SS components, could be helpful in elucidating the roles of Ced components.

As expected, the components of the Ced system are essential for DNA import. Interestingly, two components CedA1 and CedE also function in DNA export (Figure 3). *Aeropyrum pernix* K1 CedA1 pilus has the potential to transport DNA^11^. Bacterial T4SS pilus not only serves as a conduit for ssDNA transfer but also facilitates cell–cell contact via adhesin VirB5 on the tip of the pilus^19^, ^20^, ^40^, ^41^. Therefore, the participation of CedA1 and CedE in DNA export suggests that CedA1 of the receptor cell likely forms a DNA conduit that is stabilized by CedA1 and CedE proteins in the donor cell. The fact that both CedA and CedE are essential for DNA import implies that CedA and CedE may form a hetero‐oligomer on the cell membrane of the receptor cell. CedA2 is a small membrane protein containing two TM helixes like CedA1 and was proposed to form complexes with CedA and CedA1^10^. Structures of *A. pernix* K1 and *P. calidifontis* CedA1/TedC pili have been solved. However, CedA2 homolog is absent in these archaea. We tried to construct a *cedA2* deletion strain but failed; therefore, the role of CedA2 needs further investigation.

Apart from pili and pili assembly components, bacterial T4SS also have OMCC and IMCC^13^. OMCC was not found in the Ced system, possibly due to the absence of outer membrane in archaea^42^. In IMCC, VirB3 and VirB8 connect VirB6 channel on inner membrane and VirB4 ATPase ring in cytoplasm^13^, ^43^. VirB3 and VirB8 homologs are absent in archaea. Instead, about half of the CedB/CedD/VirB4 proteins in Ced systems contain TM helixes, implying direct interactions between CedA/CedE and CedB/CedD. Additionally, the distribution of CedB/CedD is complementary to that of VirB4/TedB in most *Crenarchaea*, suggesting that CedB and CedD together could function as VirB4 (Figure 4). One hypothesis is that CedB and CedD assemble into hexamer of dimers like bacterial VirB4. Homo‐oligomers of TM‐deleted CedB and CedD were obtained but purification of a stable hetero‐oligomer of TM‐deleted CedB and CedD failed (Figure 5). It was reported that VirB4 formed two side‐by‐side hexamers in the purified VirB_3‐10_ complex from pKM101 plasmid, which is quite different from its conformation in the complete pKM101 T4SS^44^. These observations suggest that full‐length CedB and CedD and whole Ced complex need to be purified in order to reveal real structural assemble and mechanism.

Bacterial T4SS encoded by conjugative plasmid is sufficient for DNA transport, while in *Sulfolobales*, the species‐specific aggregation mediated by Ups pili is also needed for DNA exchange^8^. In this study, we identified a new T4P pilin‐like protein UpsC. Deletion of other three T4Ps in *Sa*. *islandicus* REY15A did not inhibit cell aggregation while the deletion of *upsC* or *ups* operon did (Figure 6). This suggests that UpsC is an assembly component of Ups pili. In addition, although *upsC* is not close to *ups* operon in the genome, it co‐occurs with *ups* operon in *Sulfolobales* except *Acidianus*. However, the absence of Ups pili‐coding genes in other *Crenarchaea* implies that cell aggregation is not necessary for DNA transport in these species, or the function of Ups pili is replaced by other cell appendages like archaeal bundling pili found in *P. calidifontis* ^45^. Another important question to address is whether Ups pili have additional functions. In bacteria, T4P facilitates DNA uptake during natural transformation, as seen with T4aP in *Vibrio cholerae* and the Com pilus in *Streptococcus sanguinis* ^46^, ^47^. Interestingly, transjugation, a transformation‐dependent conjugation pathway in *Thermus*, utilizes T4P for DNA uptake and HerA‐like proteins, TdtA and HepA, for DNA export^48^, ^49^, ^50^. Further investigation is needed to clarify the Ups functions.

**Figure 7:**
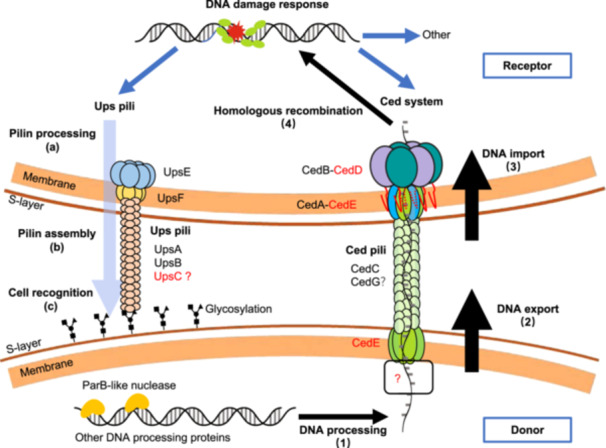
An extended model for DNA exchange in *Sa. islandicus* REY15A. After DNA damage occurs, Ups pilins are processed (a) and assembled (b) to form Ups pili, which mediate cell aggregation by recognizing species‐specific glycosylation patterns on cell surface (c). For DNA transfer, dsDNA is processed by ParB‐like nuclease (Saci_1498 homolog) and other DNA processing proteins to generate ssDNA (1). Then ssDNA is exported from CedE to CedA1 pili (2). Next, ssDNA is imported through CedA‐CedE transmembrane channel with the assistance of CedB‐CedD ATPases (3). Finally, the donor DNA is used for repairing DNA damage via homologous recombination (4). New components found in this study are labeled in red.

Based on the results of this study and previous reports, we propose an expanded DNA exchange model in *Sa. islandicus* REY15A (Figure 7). When DNA damage occurs, the transcription of DNA damage response genes, including those coding for components of the Ups and Ced systems, is induced. Ups pilins, UpsA, UpsB, and UpsC, are processed and assembled to form Ups pili which mediate cell–cell recognition with species‐specific glycosylation patterns on S‐layer^25^, ^29^, ^30^. During DNA transport, chromosomal DNA is first processed by nucleases and helicases such as ParB‐domain containing nuclease^51^. Then, the processed DNA is exported through putative CedE TM channels by unknown ATPases. Through pili conduit formed by CedA1 and possibly CedA2^11^, donor DNA is imported by CedB and CedD ATPase oligomers from CedA and CedE‐formed TM channel^10^. Finally, the DNA is used for homologous recombination repair in receptor cells. Certainly, many questions remain about the two systems, including those regarding functions of remaining UV‐inducible genes, structures of the machineries, and mechanisms underlying the formation of multi‐cell aggregates after DNA damage and discriminating receptor cells from donor cells. The DNA exchange method and results established in this study, along with advancements in structural analysis technology, are expected to facilitate the unraveling of the mystery of the fascinating Crenarchaeal DNA damage response and DNA exchange systems.

## MATERIALS AND METHODS

### Strains and culture conditions

*Sa. islandicus* REY15A (E233) (Δ*pyrEF*) and its derivatives are listed in Table S2. Cells were cultured at 75°C in MTVU media supplemented with 0.2% (w/v) D‐arabinose or sucrose as previously reported^52^. Gene knockout was conducted by transformation of knockout plasmids (Table S3) into E233S (Δ*pyrEF*Δ*lacS*) followed by selection of transformants as previously reported^53^.

### CRISPR‐Cas‐based chromosomal DNA export and import assay

Chromosomal DNA export and import assay was modified from Liu et al.^35^ Protospacers for *lacS* and *amyα* (Table S4) were cloned into the mini‐CRISPR array of pGE^53^, yielding pT*lacS* and pT*amyα*, respectively. For the DNA export assay, pT*lacS* (1 μg) was used to transform E233 (with *lacS*) cells (approximately 10^8^) by electroporation. The cells were incubated in 0.5 ml preheated medium containing mineral salts. At the same time, competent cells (10^8^) of donor strains, E233S (Δ*pyrEF*Δ*lacS*) or its derivates Δ*cedD*, Δ*cedE*, or Δ*upsC*, and so forth, were incubated in 0.5 ml preheated 2×MTV medium containing 0.4% (w/v) D‐arabinose at 75°C. After 1 h, the two cultures were mixed and incubated at 75°C for 2 h. Then, 1 ml culture was plated on the MTAV plate and cultured for 8–9 days. DNA export efficiency was defined as the CFU on the plates after mixing with the donor cells divided by CFU of E233 transformed using a non‐target plasmid. For DNA import assay, pT*amyα* (1 μg) was electro‐transformed into receptor cells, E233S (Δ*pyrEF*Δ*lacS*) or its derivatives Δ*cedD*, Δ*cedE*, or Δ*upsC*, and so forth, which contains the complete *amyα*. The cells were mixed and incubated with donor cells E233S (Δ*pyrEF*Δ*lacS*Δ*amyα*) following the same steps as for the DNA export assay. DNA import efficiency was defined as the CFU on the plates after mixing with the donor cells divided by CFU of each receptor strain transformed using a non‐target plasmid. Finally, relative DNA export and import efficiency was normalized to the efficiency of E233S.

### Phylogenetic analysis and structural prediction

For phylogenetic analysis, the homologs of CedB, CedD, and VirB4 were searched in representative *Crenarchaea* species using BLAST. The sequences were aligned using MAFFT 7.0 Webserver. Sequences of the ATPase domains were analyzed by MEGA 7.0 using the Maximum Likelihood method based on the LG+G+I+F model^54^ and annotated by iTOL (https://itol.embl.de/). The homologs of CedA, CedE, and VirB6 in archaea were searched using AlphaFold Cluster^55^, and the sequences were directly exported from these accessions (A0A2T9WR39, A0A256ZYB0, and A0A7C2LFF1). The phylogenetic tree was constructed using the same steps and based on the LG + G + F model. Structural prediction was performed by ColabFold and AlphaFold server using default parameters^56^. Structural homologs were searched using Foldseek Search^57^ and analyzed using PyMOL.

### UV treatment and RT‐qPCR analysis

The culture (30 ml) of *Sa. islandicus* E233S was treated with 100 J/m^2^ UV at an OD_600_ of 0.2 and cultured at 75°C in the dark. Samples were taken at 0, 3, 6, 12, and 24 h after treatment. Total RNA extraction, cDNA synthesis, and qPCR were performed as previously reported^58^.

### Protein expression and purification

C‐terminal His‐tagged CedBΔTM and Flag‐tagged CedDΔTM co‐expression strain E233SΔ*cedB*Δ*cedD*/piDSB‐C‐His‐CedBΔTM/C‐Flag‐CedDΔTM was cultured to an OD_600_ of 0.3 in 1 l MTV medium. Then, 10 ml of 20% (w/v) D‐arabinose was added and cultivation continued for 2 days to induce protein expression and DNA DSBs. Cells of about 4 l culture were collected and resuspended in lysis buffer (50 mM pH 8.0 Tris‐HCl, 400 mM NaCl, and 5% glycerol). After sonication, the sample was centrifuged at 4°C, 13,000*g* for 30 min, and the supernatant was loaded onto a Ni‐NTA column. The column was washed with wash buffer (20 mM imidazole in the lysis buffer) and eluted with elution buffer (400 mM imidazole in the lysis buffer). The flowthrough, which contains Flag‐tagged CedDΔTM, was further purified using anti‐Flag magnetic agarose. The eluted fractions from the nickel column and anti‐Flag magnetic agarose were concentrated by ultrafiltration. The samples were then loaded onto a Superdex 200 increase 10/300 (GE Healthcare) column for size‐exclusion chromatography using lysis buffer, and the fractions were analyzed by SDS‐PAGE, Coomassie blue staining, and Western blot.

### Cell aggregation analysis

Δ*cedA*ΔFAB, Δ*upsC*ΔFAB, Δ*upsXEFAB*ΔFAB, and the control ΔFAB, in which genes for archaellum, adhesion pili, and bindosome were deleted, were subjected to cell aggregation analysis. After UV treatment, samples were taken as described above and cell morphology was examined by microscopy under a NIKON TI‐E inverted fluorescence microscope (Nikon, Japan) in differential interference contrast. Aggregation was defined as aggregates containing at least three cells.

## AUTHOR CONTRIBUTIONS

**Pengju Wu**: Investigation (equal); methodology (equal); conceptualization (equal); data analysis (equal); writing—original draft (equal). **Mengqi Zhang**: Investigation (equal). **Yanlu Kou**: Investigation (equal). **Shikuan Liang**: Methodology (equal); writing—original draft (equal). **Jinfeng Ni**: Funding acquisition (equal); writing—review and editing (equal). **Qihong Huang**: Supervision (equal); conceptualization (equal); funding acquisition (equal); writing—original draft (equal). **Yulong Shen**: Funding acquisition (equal); supervision (equal); conceptualization (equal); writing—review and editing (equal).

## ETHICS STATEMENT

This study did not involve animals or humans.

## CONFLICT OF INTERESTS

The authors declare that the research was conducted in the absence of any commercial or financial relationships that could be construed as a potential conflict of interest.

## Supporting information

Supporting information.

## Data Availability

All data supporting the findings of this study are available within the article and the Supporting Information or from the corresponding authors upon reasonable request.
