# Supplementary material for: Identification of novel components of the Ced and Ups systems in Saccharolobus islandicus REY15A
Source: mLife. 2025 Feb 23;4(1):17–28. doi: 10.1002/mlf2.12163 (PMC11868833; doi:10.1002/mlf2.12163)
Supplement: Supplementary file 1 — Supporting information. [file MLF2-4-17-s001.docx]

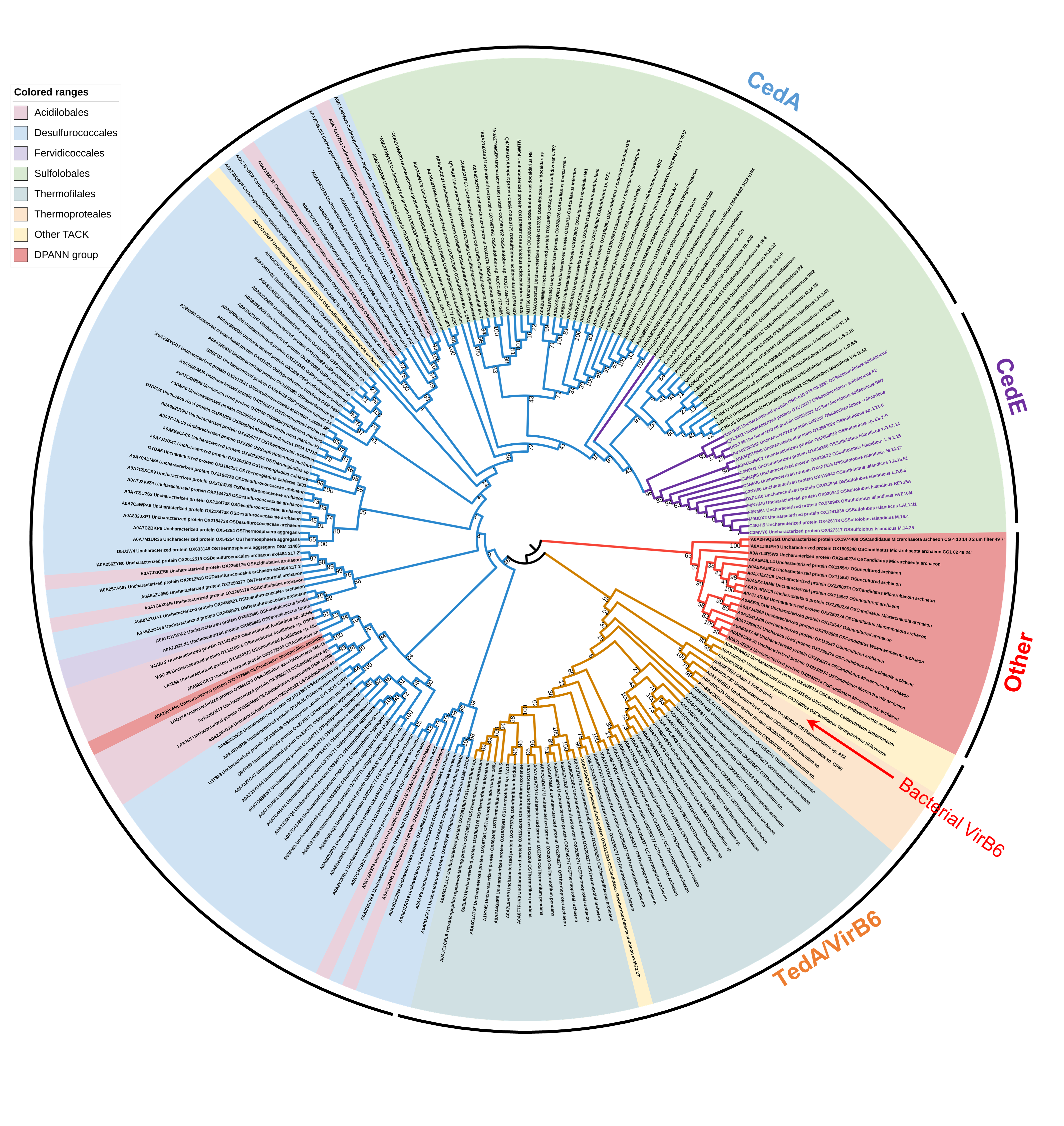
**Supplementary Figure 1. Phylogenetic analysis of archaeal CedA, CedE, TedA, and bacterial VirB6.** A total of 209 sequences from archaea and 1 from *Escherichia coli* R388 plasmid were selected, and their amino acid sequences were used to construct the Maximum Likelihood phylogenetic tree by MEGA. Bootstrap values are shown on the branch points. Archaeal protein sequences were downloaded from AFDB clusters, A0A2T9WR39, A0A256ZYB0, and A0A7C2LFF1. The bacterial VirB6 is indicated by red arrow. The UniProt ID of each protein is shown.


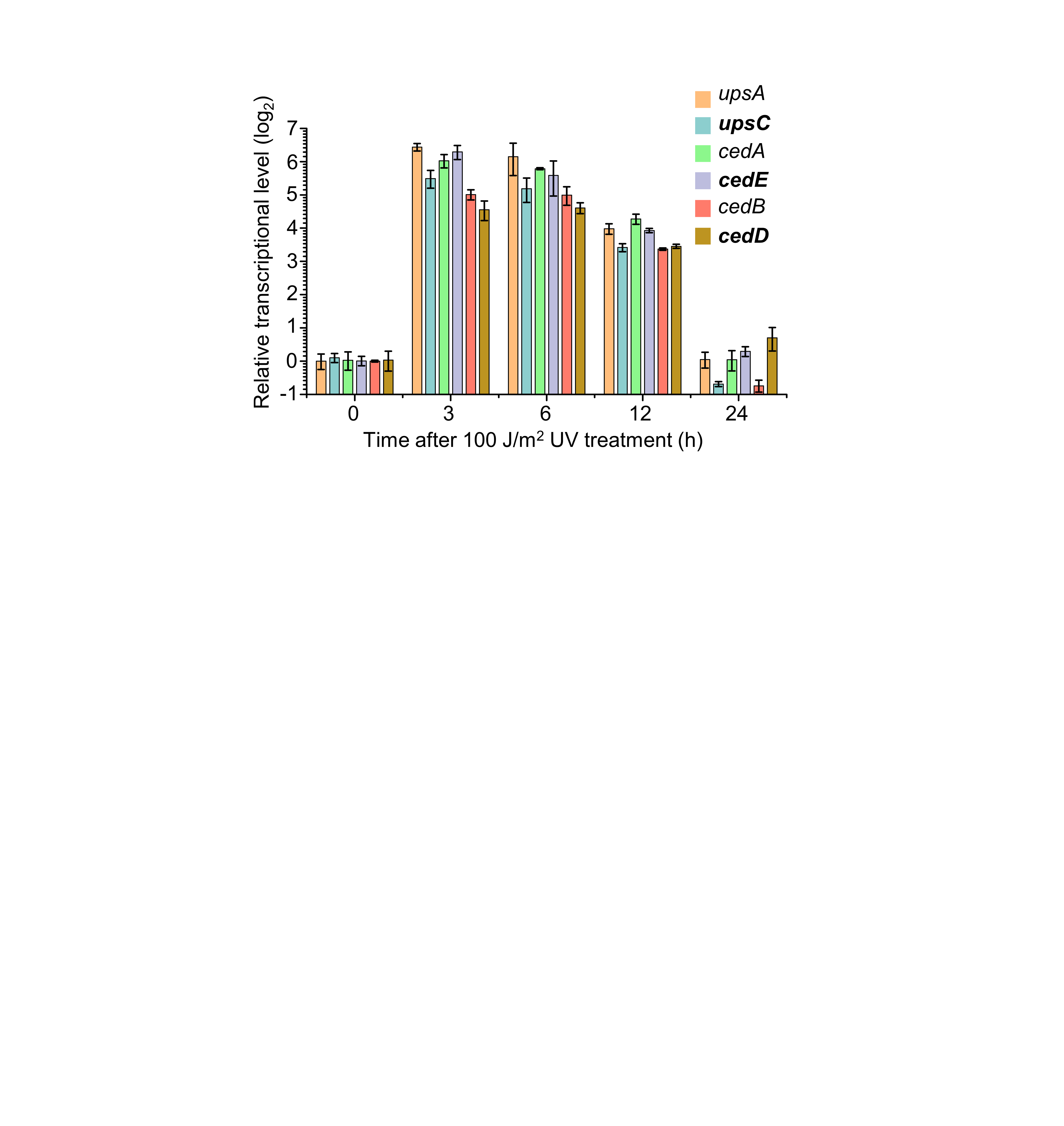
**Supplementary Figure 2. Transcriptional changes of *cedD*, *cedE*, and *upsC* after UV treatment analyzed by RT-qPCR.** Reported Ced and Ups system genes *upsA*, *cedA*, and *cedB* were used as controls. E233S culture (30 mL) was treated with 100 J/m^2^ UV. Samples were taken at 0, 3, 6, 12, and 24 hours after treatment and subjected to total RNA extraction and RT-qPCR. The transcriptional levels were normalized to the level of *tbp* based on three independent experiments.


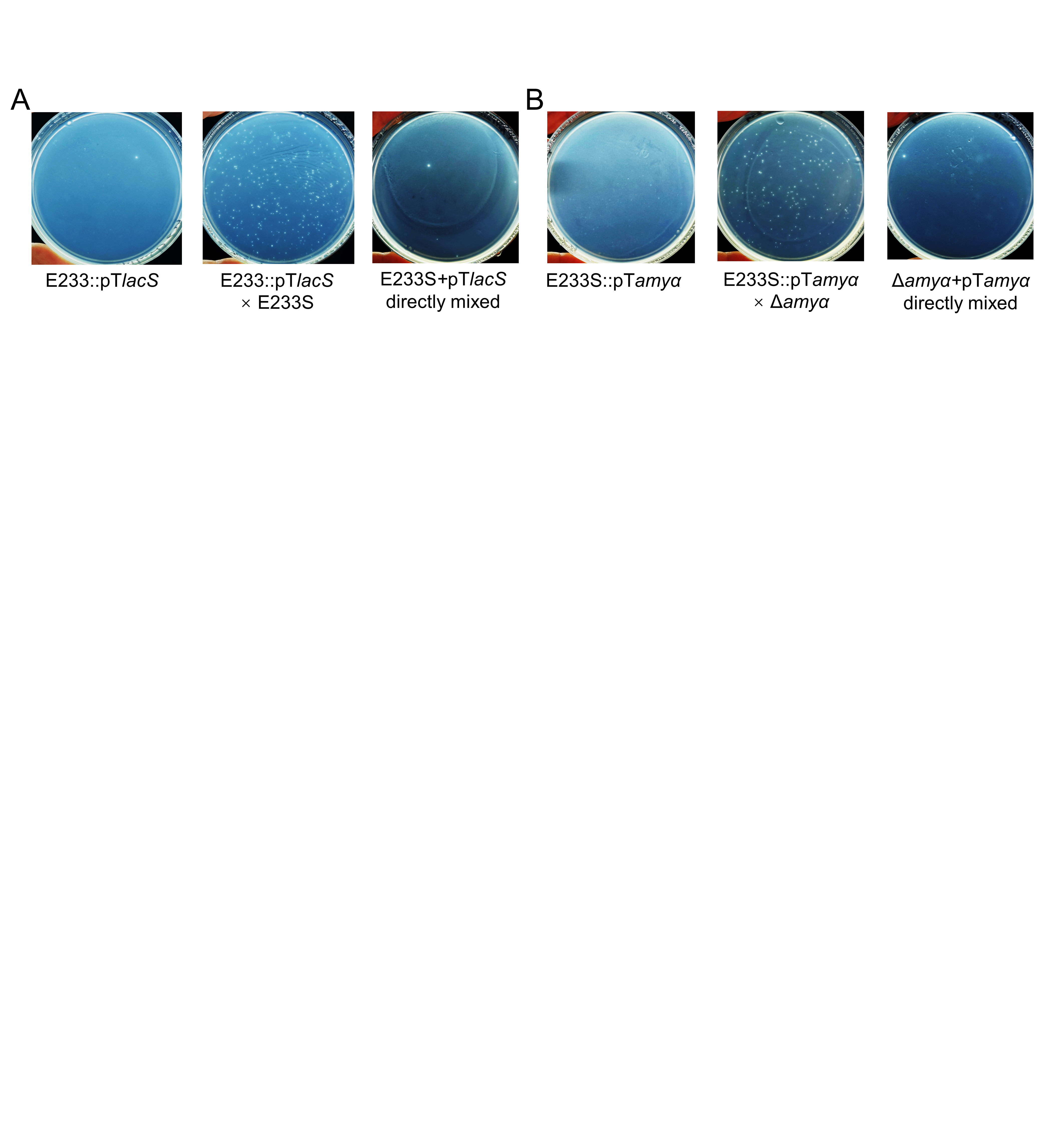
**Supplementary Figure 3. Verification of the CRISPR-Cas based chromosomal DNA export (A) and import (B) assay methods.** Left, plates showing colony formation of the receptor strains E233 and E233S transformed with target plasmids pT*lacS* and pT*amyα*, respectively. Middle, transformed receptor cells mixed with donor cells and the mixtures were incubated. Right, donor cells directly mixed with the plasmid. Representative plates are shown.


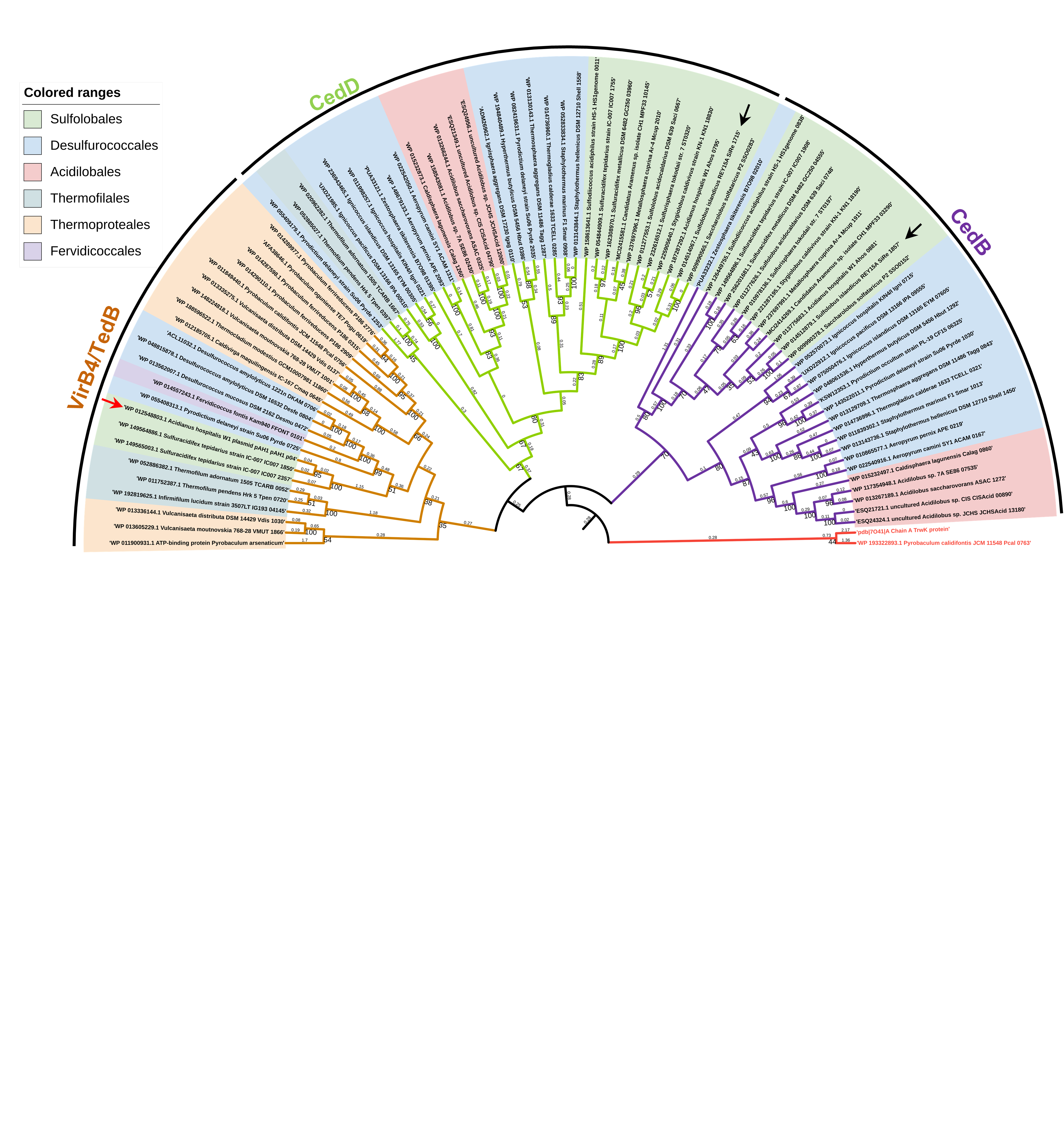
**Supplementary Figure 4. Phylogeny of CedB, CedD, and VirB4 homologs from Crenarchaeota.** A total of 86 sequences from 47 species were selected and the amino acid sequence of their ATPase domains were used to construct the Maximum Likelihood phylogenetic tree by MEGA. Bootstrap values of more than 30 are shown on each branch point and branch lengths measured in number of substitutions per site are indicated in the middle of each branches. VirB4 (PDB: 7o41) from *Escherichia coli* R388 plasmid and *Pyrobaculum calidifontis* are colored in red (bottom right). CedB and CedD from *Sa. islandicus* REY15A are indicated by black arrows and VirB4 from conjugative plasmid pAH1 of *Acidianus hospitals* W1 was indicated by red arrow.


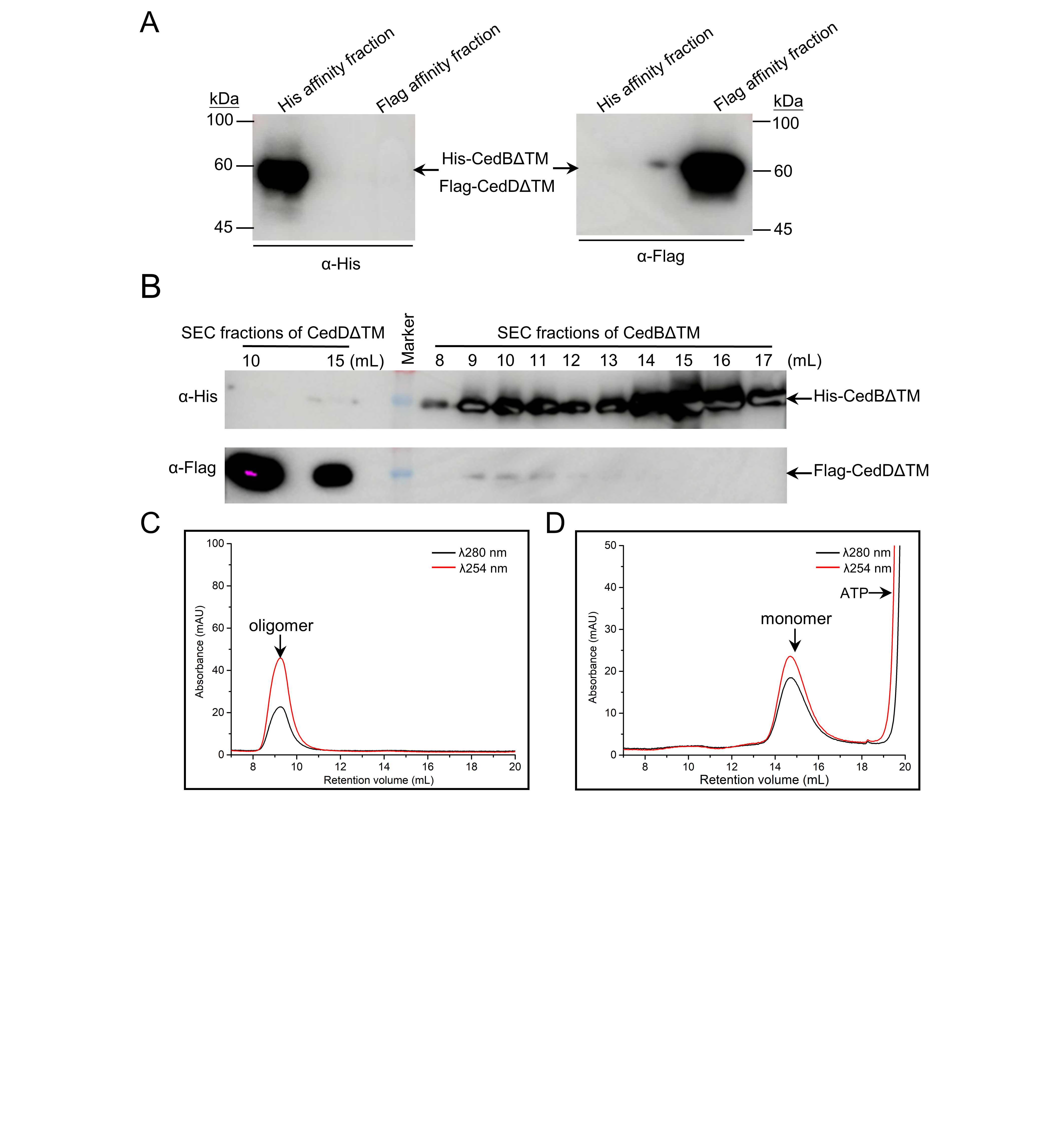
**Supplementary** **Figure 5. CedD facilitates expression and homo-oligomer formation of CedB.** (**A**) Western blot of the His-affinity (CedBΔTM) and Flag-affinity fractions (CedDΔTM) (Figure 5). CedB and CedD were detected with anti-His (left) and anti-Flag antibodies (right), respectively. (**B**) Analysis of the SEC fractions of CedBΔTM and CedDΔTM (Figure 5) by Western blot using anti-His and and-Flag antibodies. Lanes 8-17, the corresponding SEC fractions of CedBΔTM and CedDΔTM (Figure 5). (**C**) SEC analysis of the SEC purified CedBΔTM oligomer. (**D**) SEC analysis of the SEC purified CedBΔTM monomer. CedBΔTM monomer was incubated with 2.5 mM MgSO_4_ and 1 mM ATP in 75℃, 20 min before the second SEC. The arrows indicate peaks of the oligomer, monomer, and ATP.

**
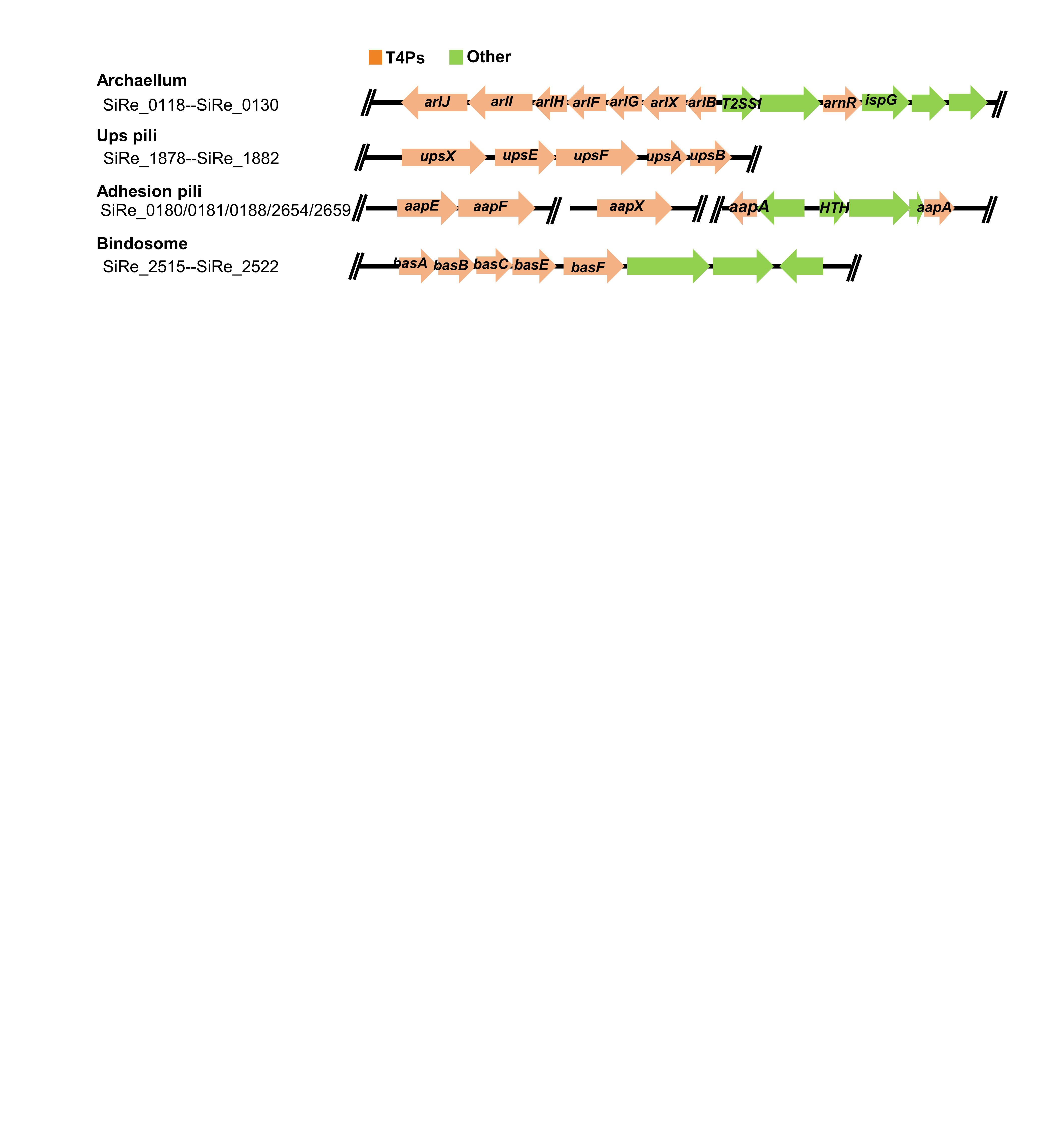
Supplementary** **Figure 6. Schematic diagram showing gene context of four Type Ⅳ pili (T4Ps) in *Sa. islandicus* REY15A.** The open reading frames of pili are shown in orange and others are shown in green.

**Supplementary Table 1.** Annotation of genes unregulated by more than 30 folds after DNA damage reagent (NQO) treatment in *Sa. islandicus* REY15A.

| Gene ID | Annotation^1,2^ | Putative functions | mRNA abundance ratio (FPKM)  ^1^ | |
| --- | --- | --- | --- | --- |
|  |  |  | E233S  -NQO | E233S  +NQO |
| SiRe_0014 | 5’-3’ exonuclease NurA-like | DNA processing | 1 | 35.557 |
| SiRe_0020 | Flg_new_2 domain-containing protein | Cell contact | 1 | 91.566 |
| SiRe_0137 | Major facilitator transporter | MFS transporter | 1 | 129.008 |
| SiRe_0187 | Coiled-coil containing protein | DNA binding | 1 | 124.001 |
| SiRe_0269 | Hypothetical protein | Unknown | 1 | 46.608 |
| SiRe_0426 | ABC transporter related membrane transporter | ABC transporter | 1 | 61.611 |
| SiRe_0589 | DUF1156 domain containing, putative adenine-specific DNA methylase | DNA processing | 1 | 108.693 |
| SiRe_0670 | SWIM (SWI2/SNF2 and MuDR) Zinc finger domain containing protein | Unknown | 1 | 54.801 |
| SiRe_0936 | Glycosyl hydrolase 15 | Unknown | 1 | 41.046 |
| SiRe_1040 | Hypothetical protein | Unknown | 1 | 38.389 |
| SiRe_1316 | CedA1 | DNA transport | 1 | 47.364 |
| SiRe_1317 | CedA | DNA import | 1 | 40.216 |
| SiRe_1318 | CedA2 | DNA transport | 1 | 45.942 |
| **SiRe_1715** | **VirB4 like ATPase/CedD** | **DNA transport** | **1** | **141.412** |
| SiRe_1717 | TFB3 | Transcriptional regulation | 1 | 105.946 |
| SiRe_1857 | CedB | DNA import | 1 | 78.300 |
| SiRe_1878 | UpsX | Unknown | 1 | 49.330 |
| SiRe_1879 | UpsE | Cell contact | 1 | 128.216 |
| SiRe_1880 | UpsF | Cell contact | 1 | 65.944 |
| SiRe_1881 | UpsA | Cell contact | 1 | 199.674 |
| SiRe_1882 | UpsB | Cell contact | 1 | 109.181 |
| **SiRe_1957** | **Prepilin domain-containing protein/UpsC** | **Cell contact** | **1** | **128.999** |
| **SiRe_2100** | **CedA paralog/CedE** | **DNA transport** | **1** | **73.161** |
| SiRe_2101 | Acyl-CoA dehydrogenase | Lipid metabolism | 1 | 31.776 |

1. Products of uncharacterized genes were annotated by sequence search, structural prediction, and homolog clustering. Protein sequences were queried using Protein BLAST. Predicted structural data were retrieved from AlphaFold Database and subjected to search using Foldseek Server. Homolog clustering was searched using AlphaFold Clusters by UniPort accession. The annotation was generated by combination of the three methods.
2. *cedD*, *cedE*, and *upsC* are highlighted in bold. Known DNA damage response genes are shown in red.

**Supplementary Table 2.** Strains used in this study

| Strain | Properties | Source |
| --- | --- | --- |
| E233 | *Sa. islandicus* REY15A Δ*pyrEF* | ^2^ |
| E233S | *Sa. islandicus* REY15A Δ*pyrEF*Δ*lacS* | ^2^ |
| Δ*amyα* | Deletion of *amyα* in E233S | This study |
| Δ*cedA* | Deletion of *cedA* in E233S | This study |
| Δ*cedA1* | Deletion of *cedA1* in E233S | This study |
| Δ*cedB* | Deletion of *cedB* in E233S | This study |
| Δ*cedD* | Deletion of *cedD* in E233S | This study |
| Δ*cedE* | Deletion of *cedE* in E233S | This study |
| Δ*cedB*Δ*cedD* | Deletion of *cedB* and *cedD* in E233S | This study |
| Δ*upsXEFAB* | Deletion of *upsXEFAB* operon in E233S | This study |
| Δ*upsC* | Deletion of *upsC* in E233S | This study |
| Δ*FAB* | Deletion of the archaellum gene cluster, *aapEaapF* operon, and the bindosome operon in E233S | This study |
| Δ*cedA*ΔFAB | Deletion of *cedA* in Δ*FAB* | This study |
| Δ*upsXEFAB*ΔFAB | Deletion of *upsXEFAB* operon in Δ*FAB* | This study |
| Δ*upsC*ΔFAB | Deletion of *upsC* in Δ*FAB* | This study |

**Supplementary Table 3.** Plasmids used in this study

| Plasmids | Properties | Source |
| --- | --- | --- |
| pGE | *Sulfolobus*-*E. coli* shuttle vector containing mini-CRISPR and *pyrEF* for CRISPR-Cas based gene editing | ^3^ |
| pSeSD | *Sulfolobus*-*E. coli* shuttle vector containing *araS*-SD promoter and MCS for proteins expressed in E233S | ^4^ |
| piDSB | Insertion of a D-arabinose inducible protospacer at mini-CRISPR array of pGE to generate DNA double stranded break | This study |
| pGE *amyα* KO | For deletion of *amyα* | This study |
| pGE *cedA* KO | For deletion of *cedA* | This study |
| pGE *cedA1* KO | For deletion of *cedA1* | This study |
| pGE *cedB* KO | For deletion of *cedB* | This study |
| pGE *cedD* KO | For deletion of *cedD* | This study |
| pGE *cedE* KO | For deletion of *cedE* | This study |
| pGE *upsXEFAB* KO | For deletion of *upsXEFAB* operon | This study |
| pGE *arl* KO | For deletion of archaellum gene cluster | This study |
| pGE *aapEaapF* KO | For deletion of *aapEaapF* operon | This study |
| pGE *bas* KO | For deletion of bindosome operon | This study |
| pSeSD-C-His-CedBΔTM | To express C-terminal His tagged CedBΔTM by pSeSD | This study |
| pSeSD-C-Flag-CedDΔTM | To express C-terminal Flag-tagged CedDΔTM by pSeSD | This study |
| pSeP*_cedB_*-C-His-CedB | To express C-terminal His-tagged CedB by replacing P*_araS_* promoter on pSeSD to P*_cedB_* promoter | This study |
| pSeP*_cedD_*-C-Flag-CedD | To express C-terminal Flag-tagged CedD by replacing P*_araS_* promoter on pSeSD to P*_cedD_* promoter | This study |
| pSeSD-C-His-CedBΔTM  /C-Flag-CedDΔTM | To co-express C-terminal His-tagged CedBΔTM and C-terminal Flag-tagged CedDΔTM by pSeSD | This study |
| pSeP*_cedB_*-C-His-CedB  /P*_cedD_*-C-Flag-CedD | To co-express C-terminal His tagged CedB and C-terminal Flag-tagged CedD by replacing P*_araS_* promoter on pSeSD to P*_cedB_* and P*_cedD_* promoters, respectively | This study |
| piDSB-P*_araS_*-C-His-CedBΔTM  /P*_araS_*-C-Flag-CedDΔTM | To co-express C-terminal His tagged CedBΔTM and C-terminal Flag-tagged CedDΔTM using P*_araS_* promoter on piDSB | This study |

**Supplementary Table 4.** Sequences of oligonucleotides used in this study.

| Oligonucleotides | Sequence (5’ to 3’) |
| --- | --- |
| *cedA* qPCR-F | CAGATCCACTACTTCAGTTTTCATCTTTT |
| *cedA* qPCR-R | CCTAAAAGGAAGTGATAATAAAAGAATACC |
| *cedE* qPCR-F | CTCTCTTACACCATAGGTACACTACTT |
| *cedE* qPCR-R | GAGGAGGCCAAAGATGTTAGCTAAT |
| *cedB* qPCR-F | GATGGAATATTGTTAGGAAAGGATCC |
| *cedB* qPCR-R | CACATTTAGCAGCGTGGATAGCC |
| *cedD* qPCR-F | TAACGGTGTCAGAGATAACCTACAC |
| *cedD* qPCR-R | TGTTATGTCCTTTACTCTTATACCTACC |
| *upsA* qPCR-F | GTCAATCCTAATACCGGACAAGCAT |
| *upsA* qPCR-R | GCCAGGTTGTAAGAGGTCGTTATTA |
| *upsC* qPCR-F | ATGCTAATCAAGTCTTTGTGGGAGTC |
| *upsC* qPCR-R | TACTTAAATGTTGCGTTGTAGACTGGTA |
| *tbp* qPCR-F | CAACAGTTACGTTAGAGCAAAGTTTGG |
| *tbp* qPCR-R | GTAACCTTGGGCTGTTCTAATCTGA |
| pT*lacS* spacer | TTTCAACAGCCTCCATATCTTTCTCCGTCAACGGTTGGAA |
| pT*amyα* spacer | GTATGCTCTTTGTGATGTTCTCCAAAAGTCTCGTAATCTA |
| piDSB spacer | TTGAGGCTAGTTCTTTGAATATTTTTGCTCTAGGATCATA |
| P*_cedB_* SphⅠ-F | TGCTGCATGCATTGAAATCCCGGGTAGAACAAAGTATT |
| P*_cedB_* MluⅠ-F | ATCGACGCGTATTGAAATCCCGGGTAGAACA |
| CedBΔTM NdeⅠ-F | TATACATATGTATAATATCGAAGATGGAGT |
| CedB noStop SalⅠ-R | ATAAGCGTCGACGAGCGTGCTACTGGCTAA |
| P*_cedD_* SphⅠ-F | TGCTGCATGCAGAGTTCAACTATGTTTTTCTTACTTCT |
| CedDΔTM NdeⅠ-F | TATACATATGCATATAAATAAATATATTCT |
| CedD Flag MluⅠ-R | GGAGACGCGTTTATTTATCGTCATCATCTTTATAGTCAGACATTTTTTTAATTTTAATT |

Restriction enzyme sites are underlined.

**Reference**

1. Sun, M., Feng, X., Liu, Z., Han, W., Liang, Y. X., & She, Q. An Orc1/Cdc6 ortholog functions as a key regulator in the DNA damage response in Archaea. *Nucleic Acids Res*. 2018;**46**:6697-6711.

2. Deng, L., Zhu, H., Chen, Z., Liang, Y. X., & She, Q. Unmarked gene deletion and host-vector system for the hyperthermophilic crenarchaeon Sulfolobus islandicus. *Extremophiles*. 2009;**13**:735-746.

3. Li, Y., Pan, S., Zhang, Y., Ren, M., Feng, M., Peng, N., et al. Harnessing Type I and Type III CRISPR-Cas systems for genome editing. *Nucleic Acids Res*. 2016;**44**:e34.

4. Peng, N., Deng, L., Mei, Y., Jiang, D., Hu, Y., Awayez, M., et al. A synthetic arabinose-inducible promoter confers high levels of recombinant protein expression in hyperthermophilic archaeon Sulfolobus islandicus. *Appl Environ Microbiol*. 2012;**78**:5630-5637.
